# The utility of prostate MRI within active surveillance: description of the evidence

**DOI:** 10.1007/s00345-021-03853-9

**Published:** 2021-12-03

**Authors:** Georgina Dominique, Wayne G. Brisbane, Robert E. Reiter

**Affiliations:** 1 Charles R. Drew University/David Geffen School of Medicine at UCLA Medical Education Program, Los Angeles, CA USA; 2 Institute of Urologic Oncology, Department of Urology, UCLA Medical Center, University of California Los Angeles, Los Angeles, CA USA; 3 Jonsson Comprehensive Cancer Center, University of California, Los Angeles, CA USA

**Keywords:** Prostate cancer, MRI, Active surveillance, PI-RADS

## Abstract

**Purpose:**

We present an overview of the literature regarding the use of MRI in active surveillance of prostate cancer.

**Methods:**

Both MEDLINE^®^ and Cochrane Library were queried up to May 2020 for studies of men on active surveillance with MRI and later confirmatory biopsy. The terms studied were ‘prostate cancer’ as the anchor followed by two of the following: active surveillance, surveillance, active monitoring, MRI, NMR, magnetic resonance imaging,  MRI, and multiparametric MRI. Studies were excluded if pathologic reclassification (GG1 →  ≥ GG2) and PI-RADS or equivalent was not reported.

**Results:**

Within active surveillance, baseline MRI is effective for identifying clinically significant prostate cancer and thus associated with fewer reclassification events. A positive initial MRI (≥ PI-RADS 3) with GG1 identified at biopsy has a positive predictive value (PPV) of 35–40% for reclassification by 3 years. MRI possessed a stronger negative predictive value, with a negative MRI (≤ PI-RADS 2) yielding a negative predictive value of up to 85% at 3 years. Surveillance MRI, obtained after initial biopsy, yielded a PPV of 11–65% and NPV of 85–95% for reclassification.

**Conclusion:**

MRI is useful for initial risk stratification of prostate cancer in men on active surveillance, especially if MRI is negative when imaging is obtained during surveillance. While useful, MRI cannot replace biopsy and further research is necessary to fully integrate MRI into active surveillance.

## Introduction

Prostate cancer is the most common internal malignancy in men [1]. Approximately 190,000 new cases and 33,000 deaths are expected during 2020, making it the second most common cause of cancer death in men [2]. However, a large number of men present with low-risk prostate cancer, and when monitored with active surveillance have less than a 2% risk of cancer-specific mortality over 10 years [3]. Prostate cancer diagnosis guided by transrectal ultrasound (TRUS) may miss higher risk prostate cancer [4]. Magnetic resonance imaging (MRI) of the prostate has emerged as a diagnostic modality to identify prostate cancer and guide biopsy. Compared to TRUS, MRI guided biopsy is more accurate for the diagnosis of prostate cancer [4].

The National Comprehensive Cancer Network, European Association of Urology and American Urological Association, recommend MRI prior to active surveillance due to improved detection of clinically significant prostate cancer (csPCa) [5–7]. The use of MRI within men already enrolled in active surveillance is still being defined. Guidelines on how to report changes in MRI among men on active surveillance are available and becoming prospectively validated [8]. MRI in active surveillance is influential as it may allow for accurate exclusion of men with csPCa and enables risk stratification to modulate the intensity of a surveillance protocol [9, 10].

The PI-RADS scoring of MRI provides a composite risk analysis for MRI, and is widely used in active surveillance protocols with good concordance [11]. In this review we evaluate the use of MRI for enrollment and predicting reclassification among men on active surveillance. Specifically, we aim to address the current literature on (1) rates of reclassification for MRI guided surveillance cohorts, (2) baseline MRI predicting reclassification, and (3), the accuracy of changes in surveillance MRI.

## Methods

### Literature search

A literature search was performed using MEDLINE^®^ and the Cochrane Library from inception to May 2020. Searches included whole field terms without quotations to maximize results. Sixteen search combinations were used with ‘prostate cancer’ as the anchor followed by two of the following: active surveillance, surveillance, active monitoring, MRI, NMR, magnetic resonance imaging,  mpMRI, and multiparametric MRI. Two reviewers (GD and WB), independently reviewed abstracts for evaluation. Due to the broad scope of this review, a single question utilizing the PICO format was not employed.

### Inclusion criteria

Only studies reporting on patients in active surveillance programs with MRI prior to confirmatory biopsy were included. Active surveillance entrance criteria were allowed to include either Grade Group 1 or low volume Grade Group 2 prostate cancer. Randomized control trials, prospective cohorts, and retrospective cohorts were included. The search was restricted to English language articles.

### Exclusion criteria

Upon full text review, studies were excluded if they included patients with Grade Group 3 or higher prostate cancer at baseline,  included patients who received cancer treatment prior to active surveillance, or represented a redundant patient population from an earlier study. Studies were excluded if they did not report a baseline PI-RADS score or an equivalent 5-point Likert scale and number of reclassifications. Meta-analyses, systematic reviews and conference abstracts were reviewed for primary references.

### Study quality

The quality of each selected article was assessed using the Grading of Recommendations Assessment, Development and Evaluation (GRADE) system [12].  The GRADE system classifies the quality of evidence into categories of high, moderate, low or very low. This tool can be applied to both randomized controlled trials and observational studies (Table 1).

**Table 1:** Study population characteristics

Author	Year	*N*	Median age	Baseline				Grade
				PSA ng/ml	PSA density ng/ml	PI-RADS distribution		
Amin [22]	2020	100	64.5	4.7 (3.4–6.6)	0.11 (0.08–0.15)	0–2	51%	High
						3	36%	
						4	12%	
						5	1%	
Chesnut [27]	2020	207	61	4.4 [3.6–5.5]	NR	0–2	40%	Moderate
						3	37%	
						4	22%	
						5	1%	
Gallagher [24]	2019	150	65.3	6.8 [6.2–7.3]	0.11 (0.08–0.17)	0–2	NR	Moderate
						3	NR	
						4	NR	
						5	NR	
Jayadevan [14]	2019	332	62.8*	4.7 [2.5–7.0]	0.08 (0.05–0.14)	0–2	31%	High
						3	42%	
						4	22%	
						5	5%	
Nougaret [34]	2017	371	60	4.7 [0.05–9.97]	NR	0–2	NR	Moderate
						3	NR	
						4	NR	
						5	NR	
Osses [26]	2020	111	66	6.8 [5.1–9.1]	0.17 (0.11–0.25)	0–2	47%	Moderate
						3	14%	
						4	32%	
						5	8%	
Pepe [32]	2020	45	66	NR	NR	0–2	NR	Moderate
						3	NR	
						4	NR	
						5	NR	
Kornberg [21]	2018	300	61.5*	NR	NR	0–2	24%	Moderate
						3	12%	
						4	44%	
						5	21%	

Study population characteristics. All included studies enrolled patients with GG1 prostate cancer. Calculations regarding reclassification were performed when patients developed ≥ GG2 prostate cancer

*Mean age

### Statistical analysis

Two by two tables for calculation of positive and negative predictive values and likelihood ratios were constructed for each study. All identified studies utilized GG1 for enrollment in active surveillance, and ≥ GG2 prostate cancer as the definition of reclassification for 2 × 2 tables (Table 2). MRI was considered positive for a PI-RADS v2 or equivalent Likert score greater than 3. For studies that did not report these data, the corresponding author was contacted to provide the missing values.

**Table 2:** MRI prediction of reclassification on active surveillance

Author	Baseline MRI likelihood ratio				Surveillance MRI likelihood ratio			
Amin [22]	PPV	38%	LR +	2.83 [1.03–7.78]	PPV	31%	LR +	5.33 [2.62–11]
	NPV	84%	LR −	0.83 [0.65–1.06]	NPV	90%	LR −	0.45 [0.26–0.78]
Chesnut [27]	PPV	NA	LR +	NA	PPV	41%	LR +	1.41 [1.21–1.64]
	NPV		LR −		NPV	85%	LR −	0.24 [0.10–0.57]
Gallagher [24]	PPV	NA	LR +	NA	PPV	23%	LR +	1.68 [1.41–2.00]
	NPV		LR −		NPV	98%	LR −	0.10 [0.01–0.70]
Jayadevan [14]	PPV	NA	LR +	NA	PPV	11%	LR +	0.86 [0.67–1.11]
	NPV		LR −		NPV	83%	LR −	1.33 [0.88–2.00]
Nougaret [34]	PPV	NA	LR +	NA	PPV	68%	LR +	5.33 [4.03–7.03]
	NPV		LR −			95%	LR −	0.13 [0.08–0.23]
Osses [26]	PPV	NA	LR +	NA	PPV	48%	LR +	1.97 [1.48–2.64]
	NPV		LR −		NPV	90%	LR −	0.25 [0.11–0.58]
Pepe [32]	PPV	NA	LR +	NA	PPV	54%	LR +	4.8 [1.88–12.00]
	NPV		LR −		NPV	91%	LR −	0.39 [0.15–0.98]
Kornberg [21]	PPV	41%	LR +	1.29 [1.15–1.45]	PPV	NA	LR +	NA
	NPV	85%	LR −	0.34 [0.19–0.62]	NPV		LR −	

All studies enrolled GG1 prostate cancer on active surveillance enrollment; 2 × 2 table values were calculated utilizing ≥ GG2 as the definition of reclassification. GRADE calculated according to template available at: https://www.gradeworkinggroup.org/. LR +  = Positive likelihood ratio, LR +  = True positive rate/false positive rate = Sensitivity/(1 − Specificity). LR − = Negative likelihood ratio, LR − = False negative rate/True negative rate = (1 − Sensitivity)/Specificity. Likelihood ratios of surveillance MRI serve to modify initial risk assessment of baseline MRI to give a probability of prostate cancer [28]

*PPV* positive predictive value, *NPV* negative predictive value

## Description of the evidence

### *Rates of reclassification in MRI selected cohorts vs. systematic biopsy cohorts*.

MRI is an effective method for ruling out clinically significant cancer at baseline. Chamie et al*.* evaluated 115 men with MRI followed by radical prostatectomy and whole-mount pathology [13]. The authors calculated a positive predictive value (PPV) of 68% and negative predictive value (NPV) of 84% for csPCa at radical prostatectomy. Similar findings were demonstrated in the PROMIS Trial which compared MRI and systematic TRUS biopsy to template transperineal biopsy, yielding a NPV of 76% and 63% for MRI and TRUS biopsy, respectively [4]. Because of the increased accuracy of MRI, men enrolled in active surveillance with baseline MRI have been hypothesized to have lower rates of reclassification at follow-up.

Jayadevan et al*.* evaluated 332 men with MRI-ultrasound fusion biopsy for active surveillance enrollment and confirmatory biopsy [14]. Within this cohort, reclassification from GG1 to ≥ GG2 prostate cancer was 13% at 1 year. Furthermore, the authors compared biopsy cores obtained from MRI targeted ROI’s and systematic samples, finding that omitting MRI biopsies missed 43% of ≥ GG2 cancer reclassifications. In the ASIST trial, Klotz et al*.*, provided level 1 evidence for the use of MRI targeted biopsy for enrollment in active surveillance [15]. In this trial 259 men were randomized between active surveillance enrollment with MRI targeted and systematic biopsy vs. systematic biopsy alone. The authors found that reclassification occurred in 9.9% of men in the MRI arm vs. 23% in the non-MRI arm at 2 years [15]. The non-MRI arm within this trial had reclassification rates similar to other large active surveillance cohorts omitting MRI at enrollment [16]. Taken as a whole, it is likely that MRI improves risk stratification for patients entering active surveillance; however, it has yet to be established if this translates into a greater number of years on active surveillance and decreased active surveillance failures. At 10 year follow-up, anywhere from 30 to 70% of patients may discontinue active surveillance [3, 17], and 59–73% seek treatment [17–19].

### Ability of positive MRI to predict reclassification among men on active surveillance

Prostate biopsy plays an essential role in active surveillance, serving as the main indicator of reclassification and prompting treatment. However, the timing and indicators for biopsy are heterogeneous among active surveillance cohorts [3, 16–19]. Elevations in PSA, PSA density, and digital rectal exam have traditionally guided clinicians in determining the frequency of prostate biopsy between scheduled intervention. Significant variations in PSA, and poor sensitivity of annual digital rectal examinations, make MRI a useful additional tool within active surveillance. High scores on PI-RADS v2 are consistently associated with clinically significant prostate cancer [20]. However, there is less known regarding whether a baseline diagnostic MRI can be used to predict future reclassification.

**Fig. 1:**
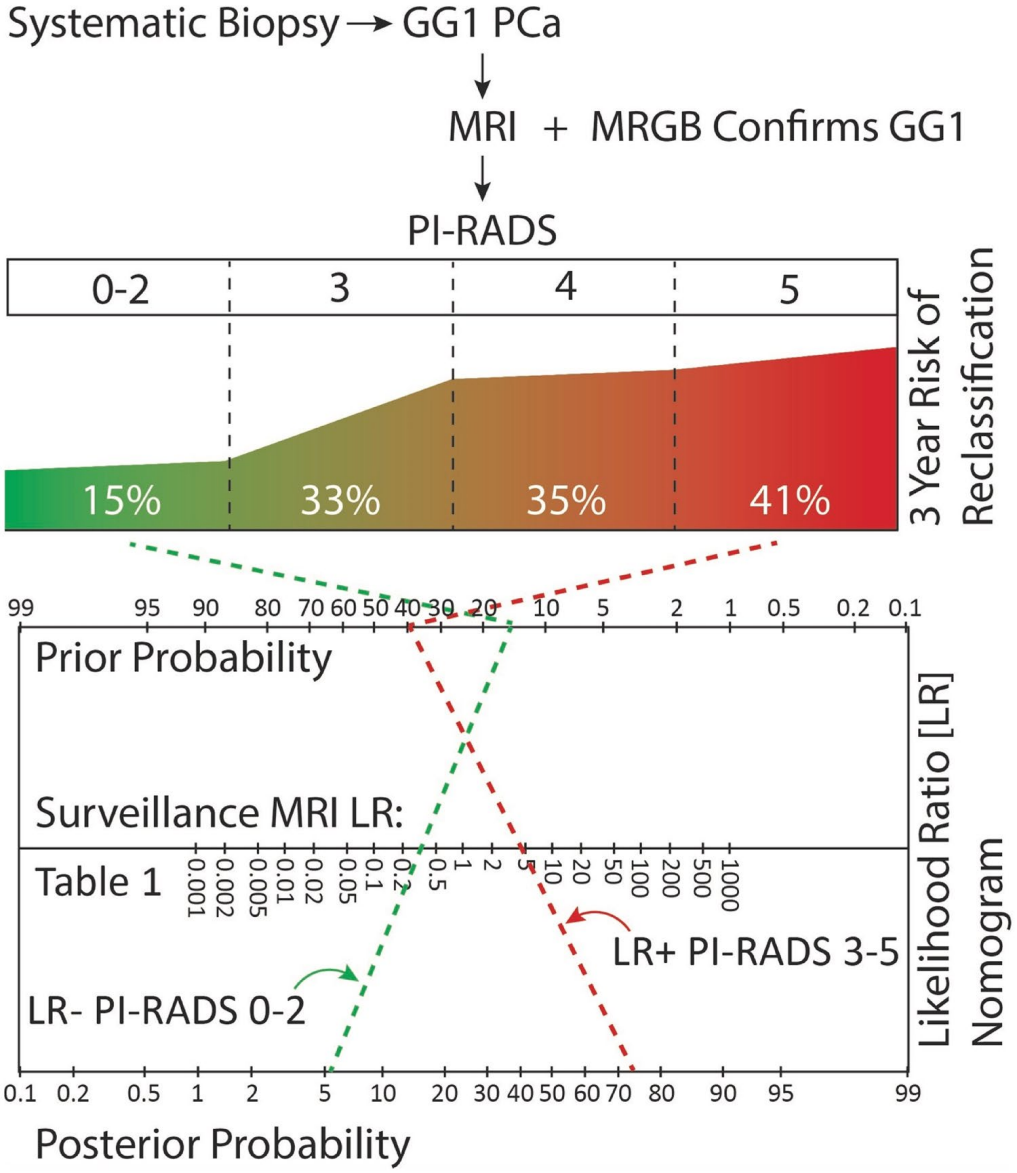
Proposed active surveillance protocol utilizing MRI for risk stratification with baseline MRI and refinement utilizing likelihood ratio calculation from Table 2. Values calculated from Kornberg et al*.* [21] and Amin et al*.* [22]

Kornberg et al., retrospectively reviewed the UCSF database, identifying 300 men with baseline MRI followed out to 5 years [21]. They evaluated the positive predictive value of baseline PI-RADS scores for reclassification at 1, 3 and 5 years. PI-RADS 5 lesions were most predictive of reclassification with PPV of 21%, 41% and 67% at 1, 3, and 5 years, respectively. PPV decreased to 13%, 33%, and 46% at the same time points when including PI-RADS 3–5 lesions. Amin et al. evaluated 100 men with MRI and transperineal template biopsy at baseline and 3 year follow-up on surveillance, finding that an initial PI-RADS score ≥ 3 yielded a positive predictive value of 38% at 3 years [22]. Both authors found the positive predictive value of MRI to be around 35–40% at 3 years (Fig. 1). Additional studies demonstrated similar positive predictive values for a baseline PI-RADS 3–5 but with higher risk of bias [23, 24].

### Ability of negative MRI to predict a negative surveillance biopsy

The negative predictive value of MRI is perhaps of more interest to patients and clinicians in active surveillance. The ability of MRI to predict a negative biopsy and thus safely avoid the associated discomfort and risks would improve current protocols. A recent meta-analysis calculated a negative predictive value of greater than 90% MRI in the biopsy naïve setting. Amin et al*.* likely performed one of the most rigorous studies evaluating the negative predictive value of MRI in active surveillance [22]. Within this cohort, 100 men were enrolled in active surveillance using a transperineal template biopsy and followed with serial MRI for 3 years. At the end of 3 years, patients were again evaluated with a template prostate biopsy. Of the 64 men with serially negative MRI, 12.5% (8/64) had clinically significant prostate cancer at 3 years. Gallagher et al*.*, followed 211 patients for a median of 4.2 years with serial MRI but less stringent entrance and exit protocol [24]. These authors found similar results among those with a negative MRI, reporting that only 12.8% (11/86) progressed to radical therapy. Overall, a negative MRI performs much better than a positive MRI in predicting biopsy results.

### Ability of changes on positive MRI to predict reclassification among men on active surveillance

Changes in MRI over time while on active surveillance represent an unsolved clinical problem. Rais-Bahrami et al*.* retrospectively evaluated 153 patients with a minimum of two MRI’s in men with small index lesions, finding minimal change over 2 years [25]. Osses et al*.* [26] evaluated radiographic progression among 111 men using the PRECISE [8] criteria. The authors found that among patients enrolled with MRI and with a positive MRI at 1 year, systematic and TRUS biopsy demonstrated reclassification in 48% of men. Among the same cohort, progression of the MRI lesion by PRECISE criteria had a positive predictive value of 41%. Similarly, Chesnut et al*.* evaluated 207 patients for MRI changes over 3 years with scheduled biopsy at baseline and 3 years [27]. The authors noted that higher PI-RADS scores were significantly associated with likelihood of reclassification; however, an increase in PI-RADS had only a 41% positive predictive value for reclassification at 3 years. Both authors concluded that PI-RADS score is an important predictor of ≥ GG2 prostate cancer but increases in PI-RADS score within a given patient on active surveillance is of uncertain utility. In general, surveillance MRI did not provide strong evidence for presence or absence of disease based on calculated likelihood ratios (Table 2) [28]. These findings are somewhat contrary to those of Felker et. al., who performed a detailed review of 49 men on active surveillance with serial MRI [29]. The authors evaluated changes in ROI volume, Apparent Diffusion Coefficient, and PI-RADS score. Among this cohort the positive predictive value of MRI change was 69%. Overall, all authors concluded that PI-RADS ≥ 3 should be investigated; however, changes between serial MRI’s may be more difficult to interpret. Studies of longer duration may address this problem definitively given the slow growth of low and intermediate risk prostate cancer. In addition, and of great importance, many authors still recommended scheduled biopsy at some frequency given the large number of reclassifications that came from systematic rather than targeted biopsy [26, 27, 29].

## Discussion

The National Cancer Care Network, American Urological Association and the European Association of Urology recommend utilization of MRI prior to enrollment of men in active surveillance [5–7]. As MRI is increasingly included among the first line diagnostic tests for elevated PSA, it is likely that patients will continue to benefit from the increasing accuracy of prostate cancer risk stratification. However, if patients are diagnosed with GG1 prostate cancer using TRUS biopsy, clinicians should consider confirmatory biopsy using MRI guidance as this is associated with higher rates of reclassification [30] (Table 2).

Once men are enrolled in active surveillance, MRI can be used to stratify the risk of reclassification. This review summarizes that the positive predictive value of baseline PI-RADS score of ≥ 3 is around 35–40% for reclassification at 3 years. Conversely, the negative predictive value for PI-RADS 1–2 is around 85% for reclassification at 3 years [21, 22] (Table 2). Risk adjusted surveillance is an area of research that deserves further attention as we found only one retrospective study studies utilizing MRI for this purpose [24]. The NCCN discourages MRI more frequently than every 12 months; however, due to the lack of evidence, guideline panels have not recommended specific intervals for MRI [6]. Additional stratification metrics such as the number of baseline positive biopsy cores, and PSA density should also be used to further refine patient follow-up [31]. Men with positive baseline MRI should be followed more closely, and we favor performing repeat MRI and biopsy within 12–24 months from baseline. Conversely, men with a negative MRI can likely undergo repeat MRI and biopsy in 24–36 months with a risk of missing about 15% of clinically significant cancer at 3 years [21, 22, 24]. In the setting of negative MRI, further reassurance is offered if the patient has a PSA density < 0.15 [14], and low PSA velocity [24, 31].

While it is concerning to have radiographic progression during active surveillance, this did not appear to consistently identify pathologic reclassification. In practice, there is often a diagnostic dilemma when a PI-RADS 5 lesion returns with only GG1 prostate cancer. In such an instance it is possible to employ both traditional and genomic biomarkers to help determine when repeat evaluation is necessary [21, 32].

The findings within this review must be interpreted within the limitations of the study. The Likelihood ratios were calculated directly or obtained from the authors; however, only Amin et al*.* and Kornberg et al*.*, had a study design which provided baseline risk of reclassification (Table 2). In addition, some studies utilized both version 1 and 2 of the PI-RADS scoring system [23, 26]. While the provided likelihood ratios are useful and allow pooling predictive data from MRI, PSA, and genomic assays, there was moderate heterogeneity in MRI. In general, surveillance MRI did not provide strong evidence for presence or absence of disease based on calculated likelihood ratios (Table 2) [28].

Overall, we found that MRI is a useful tool within active surveillance especially when used for initial risk stratification. The utility of serial MRI is most pronounced for the negative predictive value of MRI. These patients can be further risk stratified by PSA density and PSA kinetics to adjust their biopsy interval. Further refinement is necessary to incorporate MRI into active surveillance and produce individualized predictive nomograms such as those created by the PASS cohort [33].

## Data Availability

Not applicable.
